# Cyclopentenyl cytosine increases gemcitabine radiosensitisation in human pancreatic cancer cells

**DOI:** 10.1038/sj.bjc.6604287

**Published:** 2008-03-18

**Authors:** C van Bree, H M Rodermond, R Leen, J P Medema, A B P van Kuilenburg

**Affiliations:** 1 Department of Radiation Oncology, Academic Medical Centre-University of Amsterdam, Laboratory for Experimental Oncology and Radiobiology, Centre for Experimental Molecular Medicine, Amsterdam, The Netherlands; 2 Department of Genetic and Metabolic Diseases, Academic Medical Centre-University of Amsterdam, Amsterdam, The Netherlands

**Keywords:** cyclopentenyl cytosine, gemcitabine, ionising radiation, pancreatic cancer, radiosensitisation

## Abstract

The deoxycytidine analogue 2′,2′-difluoro-2′-deoxycytidine (dFdC, gemcitabine) is a potent radiosensitiser, but has limited efficacy in combination with radiotherapy in patients with pancreatic cancer due to acute toxicity. We investigated whether cyclopentenyl cytosine (CPEC), targetting the ‘*de novo*’ biosynthesis of cytidine triphosphate (CTP), could increase dFdC cytotoxicity alone or in combination with irradiation in a panel of human pancreatic cancer cells (Panc-1, Miapaca-2, BxPC-3). To investigate the role of *deoxycytidine kinase* (*dCK*), the rate-limiting enzyme in the activation of dFdC, human lung cancer cells without (dFdC-resistant SWg) and with an intact *dCK* gene (dFdC-sensitive SWp) were included. We found that CPEC (100–1000 nmol l^−1^) specifically reduced CTP levels in a dose-dependent manner that lasted up to 72 h in all cell lines. Preincubation with CPEC resulted in a dose-dependent increase in dFdC incorporated into the DNA only in dFdC-sensitive cells. Consequently, CPEC increased the effectiveness of dFdC (300 nmol l^−1^ for 4 h) only in dFdC-sensitive cells, which was accompanied by an increase in apoptosis. We also found that CPEC enhanced the radiosensitivity of cells treated with dFdC (30–300 nmol l^−1^ for 4 h). These results indicate that CPEC enhances the cytotoxicity of dFdC alone and in combination with irradiation in several human tumour cell lines with an intact *dCK* gene.

Pancreatic cancer is the fourth leading cause of cancer death worldwide with an overall 5-year survival rate less than 5%. At diagnosis, about half of all patients have unresectable, locally advanced disease, whereas 30% of the patients present with distant metastases, leaving only 20% candidates for surgery. Gemcitabine (Gemzar®, 2′2′-difluoro-2′-deoxycytidine, dFdC), a deoxycytidine analogue with a broad spectrum of antitumour activity against solid tumours, is considered to be the reference treatment for patients with locally advanced pancreatic cancer by many, but with only marginal clinical benefit (Burris *et al*, 1997). 2′,2′-Difluoro-2′-deoxycytidine has been identified as a potent enhancer of radiosensitivity of human cancer cells, including pancreatic cancer cells, which is persistent up to 72 h after the end of drug exposure (Rockwell and Grindey, 1992; Shewach *et al*, 1994; Lawrence *et al*, 1996, 1997; Buchsbaum *et al*, 2002). Several phase I and II studies have investigated concurrent dFdC and radiotherapy, but acute gastrointestinal toxicity was encountered with standard doses of dFdC and radiotherapy, which depended on the irradiated volume (Crane *et al*, 2001; Wilkowski *et al*, 2006; Murphy *et al*, 2007). Either with a reduction of the dFdC dose or the radiation dose or the volume, concurrent chemoradiotherapy appears feasible. However, this will likely reduce the efficacy of treatment whereas an increase in efficacy of the combined treatment for local tumour control is clearly required (Wilkowski *et al*, 2006; Yip *et al*, 2006; Murphy *et al*, 2007; Yamazaki *et al*, 2007).

Several approaches, based on increasing the anabolism of dFdC, have been described to enhance its effectiveness (Duxbury *et al*, 2004; Giovannetti *et al*, 2004; Verschuur *et al*, 2004; Bierau *et al*, 2006). 2′,2′-Difluoro-2′-deoxycytidine is activated by intracellular phosphorylation to its active metabolites dFdC diphosphate and dFdC triphosphate. The initial phosphorylation to dFdC-monophosphate is performed by *deoxycytidine kinase* (*dCK*) and is the rate-limiting step. The activity of *dCK* is feedback-inhibited by dCTP present in cells. The ‘*de novo*’ pathway for the synthesis of both cytidineribonucleotides and cytidinedeoxyribonucleotides is mediated by the enzymes *CTP synthetase* (*CTPs*). This pathway has been reported to be upregulated in solid tumours (Kizaki *et al*, 1980) and is therefore an attractive target for increasing the therapeutic ratio of dFdC and radiotherapy.

Cyclopentenyl cytosine (CPEC, NSC 375575) is a cytidine analogue which, in its active 5′-triphosphate form, is a noncompetitive inhibitor of *CTPs* leading to depletion of both cytidine pools and deoxycytidine pools (Kang *et al*, 1989; Verschuur *et al*, 2004; Bierau *et al*, 2006). Consequently, mRNA and protein levels as well as the activity of *dCK* are elevated (Bierau *et al*, 2006), which enhance the effectiveness of dFdC in human leukaemia (Verschuur *et al*, 2004) and human neuroblastoma cells (Bierau *et al*, 2006). However, to date there is no information available whether CPEC could enhance dFdC effectiveness in human pancreatic tumour cell lines nor on the interaction between dFdC and radiation. In this report, we show that CPEC strongly enhances the effectiveness of dFdC alone and in combination with radiation in three human pancreatic tumour cell lines. We incorporated a dFdC-sensitive and a dFdC-resistant human nonsmall cell lung carcinoma (NSCLC) cell line (SWp and SWg respectively, van Bree *et al*, 2002) to show that these CPEC-induced effects can also be obtained in other human solid tumour cell lines and requires the presence of *dCK* activity.

## MATERIALS AND METHODS

### Drugs and chemicals

Leibovitz-15 medium (L-15), DMEM, RPMI with HEPES and PSG (100 × stock of 10 000 U ml^−1^ penicillin, 10 mg ml^−1^ streptomycin and 20 mmol l^−1^ glutamine) were purchased from GIBCO-BRL (Paisley, Scotland), cell proliferation kit II from Roche (Mannheim, Germany), dFdC from Eli Lilly (Nieuwegein, The Netherlands) and [^3^H]-dFdC (^14^Ci mmol^−1^) from Moravek Biochemicals (BREA, CA, USA). Cyclopentenyl cytosine (NSC 375575) was obtained from the Developmental Therapeutics Program, National Cancer Institute (Bethesda, MD, USA). All nucleotide standards were obtained from Sigma Chemicals (Zwijndrecht, The Netherlands). All other chemicals were of analytical grade and commercially available.

### Cell cultures

Human pancreatic cell lines Panc-1, Miapaca-2 and BxPC-3 (American Type Culture collection, Manassas, VA, USA) were grown as monolayers in DMEM (Panc-1 and Miapaca-2) or RPMI (BxPC-3) supplemented with 10% heat-inactivated fetal bovine serum (FBS) and with PSG at 37°C at 5% CO_2_. The human NSCLC SWp and its dFdC-resistant variant SWg have been described (van Bree *et al*, 2002; Jordheim *et al*, 2004). These cells were grown as monolayers in L-15 medium supplemented with 10% heat-inactivated FBS and PSG at 37°C without additional CO_2_. All cell lines were passaged twice weekly to ensure exponential growth.

### Drug treatment and irradiation

Cells were seeded (8500 cells per cm^2^) in Petri dishes and were allowed to attach overnight. Various doses of CPEC and dFdC were added from freshly prepared 100 × stock solutions in sterile phosphate-buffered saline without refreshing the medium. Cells were irradiated with *γ* rays from a ^137^Cs source at a dose rate of approximately 0.7 Gy min^−1^.

### Extraction and analysis of nucleotides and detection of radiolabelled dFdC metabolites

For the analysis of the effects of CPEC alone on nucleotide triphosphate levels and for the detection of radiolabelled metabolites of dFdC after a 4 h incubation with 300 nM [^3^H]-dFdC, cells were extracted with 200 *μ*l of ice-cold 0.4 M perchloric acid for 10 min on ice with intermittent scraping with a disposable cell scraper. The resulting suspension was centrifuged at 10 000 **g** at 4°C for 5 min. Supernatant was removed, neutralised with K_2_CO_3_ and used for HPLC analysis. Nucleotide profiles were determined by ion-exchange HPLC, using a Whatman (Clifton, NJ, USA) Partisphere SAX 4.6 × 125 mm column (5 *μ*m particles) and a Whatman 10 × 2.5 mm AX guard column. The pellet obtained after perchloric acid precipitation was taken up in 300 *μ*l of 0.2 M NaOH and precipitated again by adding an equal volume of 1.2 M perchloric acid. The protein- and DNA-containing fraction was obtained by centrifugation and the pellet dissolved in a final volume of 200 *μ*l NaOH. The protein content was determined using bicinchoninic acid solution containing 0.1% CuSO_4_ using bovine serum albumine as a standard. Radioactivity was detected on-line with a Radiometric 525TR Flow Scintillation Analyser with a 500 *μ*l TR-LSC cell (Packard, Meriden, CT, USA) using Ultima Flo AP (Packard, Dowers Grove, IL, USA) at an effluent-to-scintillation fluid ratio of 1 : 1. Radioactivity of the protein pellet was measured on a *ß* counter (Bierau *et al*, 2003).

### Clonogenic and proliferation assay

Cells were harvested at different time points and different treatments, kept on ice, counted, diluted and sparsely plated for standard clonogenic assay (Franken *et al* (2006); plating efficiencies for Panc-1, Miapaca-2, SWp and SWg cells were 0.62±0.07 (mean±s.e.), 0.35±0.08, 0.89±0.10 and 0.80±0.08 respectively). In parallel experiments, the treated cells were plated for proliferation assay at higher densities (8500 cells per cm^2^) in 6- or 96-well plates to allow reutilisation of dFdC from dying cells (Rockwell and Grindey, 1992). Six-well plates were fixated after 6–10 days with 6% glutaraldehyde and stained with crystal violet. Wells were scanned with HP Scanjet 5300C using HP Precision Scan-software and HP Intelligent Scanning Technology (version 3.4). Proliferation in 96-well plates was determined by the cell proliferation kit II according to the recommendations of the manufacturer (Roche Diagnostics GmbH, Mannheim, Germany).

### Apoptosis by DNA fragmentation assay

A flow cytometric method was used for measuring the percentage of apoptotic nuclei after propidium iodide staining in hypotonic buffer, and thereby assessing apoptosis of specific cell populations in heterogeneous tissues (Nicoletti *et al*, 1991). Both detached and attached cells were harvested, pelleted and counted. From each sample, 2 × 10^5^ cells were resuspended in 100 *μ*l of Nicoletti buffer (0.1% sodium citrate, 0.1% Triton X-100, 50 *μ*g ml^−1^ propidium iodide, dissolved in demi water) and stored for 24 h at 4°C. Flow cytometry was performed with FACScan cytometer (BD, San Jose, CA, USA).

### Statistical analysis

Differences in radiosensitivity were analysed using SPSS (Chicago, IL, USA) statistical software by means of a fit of the data by a weighted, stratified, linear regression, according to the linear-quadratic formula (Franken *et al*, 2006). All other differences between experimental groups were analysed by the two-tailed Student's *t*-test assuming equal or unequal variances using Microsoft Excel.

## RESULTS

### CPEC depletes cellular CTP levels and increases the anabolism of dFdC

**Figure 1:**
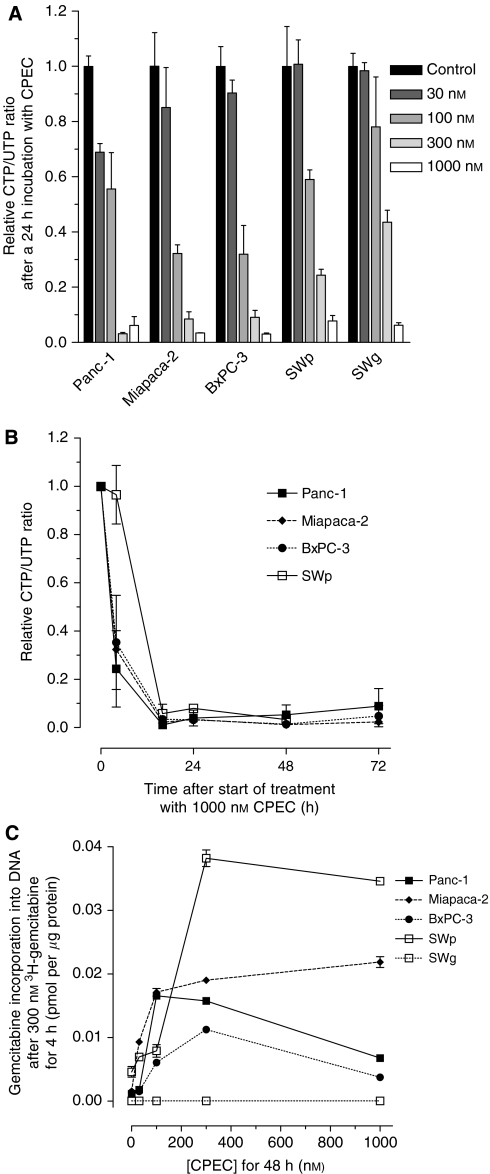
The effects of CPEC with respect to CTP depletion and to the anabolism of dFdC in human pancreatic carcinoma (Panc-1, Miapaca-2 and BxPC-3) and NSCLC cells (SWp and its dFdC-resistant variant SWg). Cellular CTP/UTP ratios relative to untreated controls are shown as means with standard errors of at least three separate experiments for the dose dependency at 24 h after CPEC (**A**) and for the kinetics after exposure to 1000 nM CPEC (**B**). Incorporation of dFdC into DNA as a function of CPEC dose for 48 h preincubation are shown as means with standard errors of at least three separate experiments (**C**).

Three widely used human pancreatic carcinoma cell lines, Panc-1, MiaPaca-2 and BxPC-3, in which dFdC-induced radiosensitisation has been described (Lawrence *et al*, 1996, 1997; Buchsbaum *et al*, 2002), were selected for this study. First, the ability of CPEC to specifically deplete CTP pools was investigated (Figure 1A and B). In all three cell lines, CPEC specifically reduced CTP/UTP ratios in a dose-dependent manner (Figure 1A) without prominent effects on ATP levels (varying between 0.8 and 1.5 for all data points). Importantly, the decrease in CTP levels was not dependent in the presence of a wild-type *dCK* activity, as the human NSCLC cell line SWg, which has a disrupted *dCK* gene (van Bree *et al*, 2002; Jordheim *et al*, 2004), showed similar CTP depletion as compared to its parental cell line SWp. The depletion of CTP was already detected at 4 h of exposure to 1000 nM CPEC and was almost complete at 16 h (Figure 1B). The reduction of CTP levels by CPEC was maintained in all cell lines up to 72 h. We next determined the ability of CPEC to enhance the anabolism of dFdC (Figure 1C). The dFdC-resistant cell line SWg served as a control and confirmed the necessity of normal *dCK* activity for incorporation of dFdC. In all other cell lines, an increase of ^3^H-dFdC incorporation into the DNA was observed after preincubation of the cells for 48 h with CPEC doses as low as 30 nM. For the dFdC-sensitive cell lines, a maximal increase in dFdC incorporation of 10- to 15-fold was observed at 100–300 nM CPEC.

### CPEC increases the efficacy of dFdC

**Figure 2:**
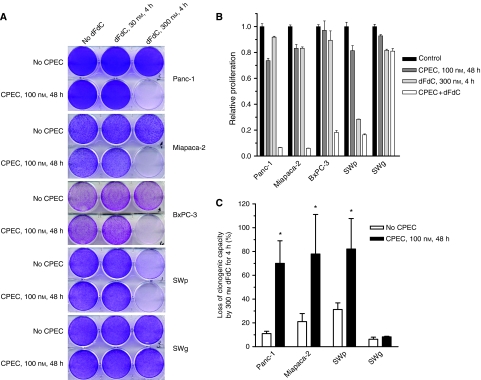

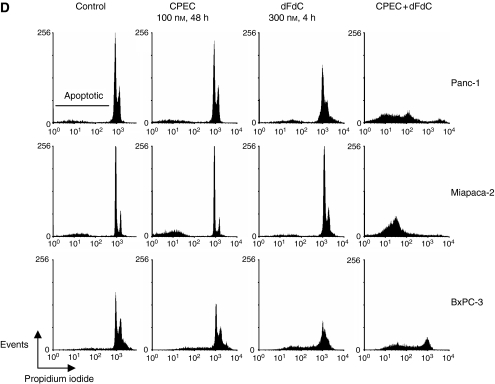
The effects of preincubation with CPEC on the effects of dFdC with respect to cell proliferation (**A** and **B**), loss of clonogenic capacity after correction for the cytotoxicity of CPEC alone ((**C**) Panc-1: 0.71±0.12; Miapaca-2: 1.00±0.06; SWp: 1.07±0.08; SWg: 0.85±0.26) and induction of apoptosis (**D**) in human pancreatic carcinoma (Panc-1, Miapaca-2 and BxPC-3) and NSCLC cells (SWp and its dFdC-resistant variant SWg). Representative examples (**A** and **D**) or means with standard errors of at least three separate experiments (**B** and **C**, ^*^P<0.05) are shown.

As an increased anabolism of dFdC has been shown to enhance the antiproliferative effect of dFdC (Verschuur *et al*, 2004; Bierau *et al*, 2006), we determined the treatment efficacy of CPEC and dFdC in our cell panel (Figure 2). Preincubation for 48 h with 100 nM CPEC markedly increased the growth inhibitory effect of dFdC, but only at high concentration (Figure 2A and B). This was also observed for exposures to higher doses of CPEC (up to 1000 nM, data not shown). As expected from Figure 1C, CPEC could not increase the sensitivity to dFdC of the dFdC-resistant SWg cells. In addition to the antiproliferative effects of the combined treatment, we determined the clinically more relevant effects on cell survival by clonogenic assay and on apoptosis. After correction for the toxicity of CPEC alone, we observed that the combined treatment of dFdC-sensitive pancreatic cell lines that display clonogenic growth is significantly more effective than dFdC alone in reducing survival (Figure 2C). We noted that different incubation periods (24–72 h) with CPEC did not induce significant differences in its cytotoxicity nor in dFdC cytotoxicity in Panc-1 and SWp cells. Using DNA fragmentation after treatment as an indicator of apoptosis, we observed that CPEC markedly increased dFdC-induced apoptosis in the three human pancreatic tumour cell lines (Figure 2D). This increase in apoptosis likely contributes to the improved efficacy of dFdC.

### CPEC increases dFdC-induced radiosensitisation

**Figure 3:**
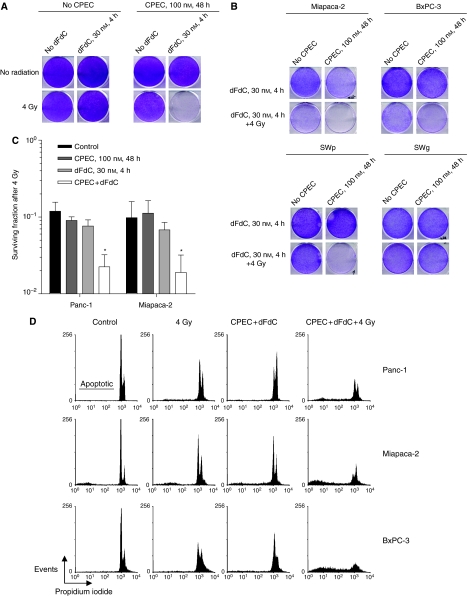
Treatment efficacy of preincubation with CPEC, a nonradiosensitising dose of dFdC and irradiation in human pancreatic carcinoma cells (Panc-1, Miapaca-2, BxPC-3) and NSCLC cells (SWp and its dFdC-resistant variant SWg). Cyclopentenyl cytosine enhanced the efficacy of dFdC combined with radiation with respect to cell proliferation in Panc-1 (**A**) and Miapaca-2, BxPC-3, SWp, but not in SWg cells (**B**). Clonogenic survival after radiation (**C**) is shown for Panc-1 and Miapaca-2 cells after correction of the toxicity of either treatment without irradiation (Panc-1: CPEC, 0.71±0.12; dFdC, 1.01±0.03; CPEC+dFdC, 0.40±0.03; Miapaca-2: CPEC, 1.00±0.06; dFdC, 0.52±0.20; CPEC+dFdC, 0.54±0.08). Induction of apoptosis (**D**) is shown for Panc-1, Miapaca-2 and BxPC-3. Representative examples (**A**, **B** and **D**) or means with standard errors are shown of at least three separate experiments (**C**, ^*^P<0.05).

**Figure 4:**
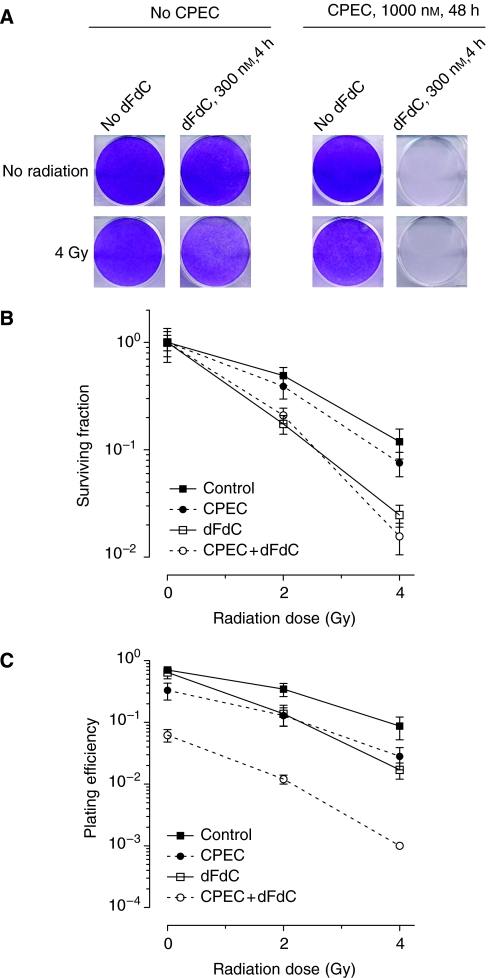
Preincubation with CPEC (1000 nM for 48 h) additively enhances dFdC-induced (300 nM for 4 h) radiosensitisation in human pancreatic carcinoma Panc-1 cells. Representative example of a proliferation assay (**A**) or means with standard errors of at least three separate experiments are shown for clonogenic survival after correction for the toxicity of either treatment alone (**B**, Panc-1: CPEC, 0.53±0.16; dFdC, 0.89±0.13; CPEC+dFdC, 0.10±0.02) or for plating efficiency without the correction (**C**). Significant radiosensitisation was observed for dFdC alone and for CPEC combined with dFdC (**B**, *P*<0.001), but not after CPEC alone.

As dFdC is a well-known radiosensitiser of human cancer cells including pancreatic carcinoma cells (Rockwell and Grindey, 1992; Shewach *et al*, 1994; Lawrence *et al*, 1996, 1997; Buchsbaum *et al*, 2002), we investigated the effects of CPEC on dFdC-induced radiosensitisation in our cell panel (Figure 3). In most clinical trials investigating concurrent application of dFdC and radiotherapy, the dFdC dose is reduced to circumvent acute gastro-intestinal toxicity (Crane *et al*, 2001; Wilkowski *et al*, 2006), which likely reduces treatment efficacy. We therefore studied the interaction with radiation of a lower dose of dFdC (30 nmol l^−1^ for 4 h), which by itself does not induce radiosensitisation (Figure 3). In proliferation assays, CPEC, dFdC and radiation alone hardly affected the growth of Panc-1 cells (Figure 3A). This dFdC dose did not induce radiosensitisation in proliferation or clonogenic assays (Figure 3C). Although CPEC was not able to enhance the efficacy of this lower dFdC dose, it could clearly inhibit proliferation when combined with dFdC as well as radiation. This effect was also observed in the other dFdC-sensitive cells, but not in dFdC-resistant SWg cells (Figure 3B). Clonogenic survival analysis demonstrated that CPEC could significantly increase the radiosensitivity of Panc-1 and Miapaca-2 cells that were also treated with dFdC (Figure 3C). This increased efficacy of dFdC and radiation induced by CPEC was again accompanied by an increased apoptosis in all three human pancreatic cell lines (Figure 3D). Subsequently, we investigated whether CPEC influenced the interaction between dFdC and radiation in a higher dose of dFdC (300 nmol l^−1^ for 4 h), which may represent the clinical setting in which a full dFdC dose can be given. In Panc-1 cells, CPEC was again able to completely inhibit proliferation when combined with dFdC (Figure 4A). A further inhibition of proliferation by the addition of radiation could not be detected. To investigate the possible influence of CPEC with or without dFdC on radiosensitivity, clonogenic assays were performed (Figure 4B). We observed that dFdC alone induced significant radiosensitisation in Panc-1 cells (*P*<0.001), but that CPEC alone did not. The radiation dose survival curves of CPEC combined with dFdC and that of dFdC alone are similar, indicating that in the combined treatment, dFdC-induced radiosensitisation was still present. Similar observations were made for MiaPaca-2 and SWp cells and for preincubation for 48 h with 100 nmol l^−1^ of CPEC (data not shown). As we demonstrated a significant increase in efficacy of dFdC by preincubation with CPEC (Figure 2C), the plating efficiency, that is, the number of surviving colonies relative to the number of cells plated, after the various treatments is shown (Figure 4C). The combined treatment of CPEC and dFdC combined with radiation is clearly the most effective in reducing cellular survival of Panc-1 cells.

## DISCUSSION

Cyclopentenyl cytosine in its triphosphate form is an antagonist of *CTP synthetase*, which catalyses the conversion of UTP into CTP. In this paper, we show for the first time that CPEC is able to induce specific depletion of CTP levels in human pancreatic carcinoma and NSCLC cells, which markedly sensitised these cells for treatment with dFdC alone and in combination with radiation. This was achieved at clinically relevant doses of CPEC that were previously shown to decrease CTP levels in leukaemic samples of 85 adult and paediatric patients (Verschuur *et al*, 2000) and reduced CTPs activity in bone marrow mononuclear cells of patients treated with CPEC (Politi *et al*, 1995). Cyclopentenyl cytosine was shown to be active against leukaemia, glioblastoma, neuroblastoma and colon carcinoma (Moyer *et al*, 1986; Viola *et al*, 1995; Verschuur *et al*, 2002; Bierau *et al*, 2003). In humans, CPEC has been studied in a phase I clinical trial in adults with solid tumours (Politi *et al*, 1995); 26 patients suffering from predominantly colon carcinoma were treated every 3 weeks with increasing doses of CPEC, ranging from 1 to 5.9 mg m^−2^ h^−1^ for 24 h (total of 87 cycles). Only mild toxicity was observed in patients with steady-state plasma concentrations below 1.5 *μ*M (3.0 mg m^−2^ h^−1^). The most severe toxicity was cardiovascular: six episodes of hypotension occurred in five patients who had been treated with doses ranging from 3.0 to 4.7 mg m^−2^ h^−1^. Two patients treated with 4.7 mg m^−2^ h^−1^ experienced fatal hypotension, which has never been fully explained. The conclusion from these results was, therefore, not to proceed clinically with CPEC as a single agent for solid tumours, but to investigate the exploitation of its targeting effect on CTPs.

Targeting of the *de novo* pathway for the synthesis of nucleotides by RNAi against a subunit of ribonucleotide reductase has proven to be an effective strategy to enhance the effectiveness of dFdC in a xenograft pancreatic model (Duxbury *et al*, 2004). Recently, CPEC has been shown to enhance dFdC effectiveness in human leukaemia (Verschuur *et al*, 2004) and in human neuroblastoma cells (Bierau *et al*, 2006). Similar to our findings in human pancreatic carcinoma and NSCLC cells, CPEC enhances the incorporation of dFdC and other nucleotide analogues such as cytarabine into the DNA (Verschuur *et al*, 2002, 2004; Bierau *et al*, 2003, 2006). A decrease in the feedback inhibition of dCTP on *dCK* was suggested to be the underlying mechanism (Verschuur *et al*, 2004; Bierau *et al*, 2006). But even a 2-h exposure to CPEC, which is probably too short to influence *dCK* activity, already increased the anabolism of cytarabine (Verschuur *et al*, 2002). The specific depletion of CTP by CPEC may therefore be more important in the enhanced anabolism of dFdC and cytarabine. Another drug, which depletes cellular nucleotide pools, is the multitargeted antifolate pemetrexed (Giovannetti *et al*, 2004). Although less specific as compared to CPEC, pemetrexed has also been shown to synergistically interact with dFdC in human pancreatic cancer cells (Giovannetti *et al*, 2004). To our knowledge, these strategies have not been applied in combination with radiation, which has been shown to alleviate pain in patients with locally advanced pancreatic cancer (Ceha *et al*, 2000). Although there is insufficient evidence to recommend chemoradiation in patients with locally advanced inoperable pancreatic cancer as a superior alternative to dFdC alone (Yip *et al*, 2006), an increase in treatment efficacy is clearly required for this patient group (Wilkowski *et al*, 2006; Yip *et al*, 2006; Murphy *et al*, 2007; Yamazaki *et al*, 2007).

Both *in vitro* and *in vivo*, dFdC is a potent enhancer of the cytotoxic effects of ionising radiation (Rockwell and Grindey, 1992; Shewach *et al*, 1994; Lawrence *et al*, 1996, 1997; Buchsbaum *et al*, 2002). Our results show that the increased incorporation of dFdC by CPEC clearly enhanced the efficacy of dFdC, which was shown for proliferation as well as for clonogenic survival. An elevated induction of apoptosis is likely to be one of the underlying mechanisms. For the interaction with radiation, an additive enhancement was observed if a radiosensitising dose of dFdC was used and a more than additive interaction if a nonradiosensitising dose of dFdC was used. This is in agreement with earlier findings that radiation enhancement by dFdC increases with increasing dFdC dose, but with an optimum, likely to be due to its inhibitory effect on *dCK* at higher concentrations (Shewach *et al*, 1994). Recently, the simultaneous alteration of the *de novo* and salvage pathway to the deoxynucleoside triphosphate pool by (E)-2′-deoxy-(fluoromethylene)cytidine and zidovudine has also been shown to increase the radiosensitivity of human colon cancer cells *in vitro* (Coucke *et al*, 2007). Our survival data were obtained using a standard clonogenic assay in which sparsely plated cells are used (Franken *et al*, 2006). When similar cell numbers were plated in higher density, the interaction of CPEC, dFdC and radiation appears to be more impressive. This phenomenon, called reutilisation, has been noted earlier for dFdC and has been suggested to be the cause for the efficacy of dFdC in solid tumours (Rockwell and Grindey, 1992; Haveman *et al*, 1995). These observations were verified in all three human pancreatic carcinoma cells as well as in human NSCLC cells. Moreover, the importance of an intact *dCK* gene in this interaction was demonstrated by the dFdC-resistant variant of the human NSCLC SWp (van Bree *et al*, 2002; Jordheim *et al*, 2004). The observed differences in dFdC incorporation between the cell lines used in this study suggest that they differ in *dCK* activity. Cyclopentenyl cytosine clearly enhanced the dFdC incorporation in cells with an intact *dCK* gene, which may be related to an enhanced *dCK* activity (Bierau *et al*, 2006). This would be in agreement with the finding that the *dCK* activity correlates with dFdC-induced radiosensitisation (Gregoire *et al*, 2002).

In conclusion, we report that CPEC in a low, clinically achievable and nontoxic dose increases dFdC effectiveness as well as its radiosensitising effect in human pancreatic carcinoma cells. Since heterogeneous chemotherapeutic distributions are inherent to solid tumours (Jain, 2001), the combination of CPEC, dFdC and radiation appears to be a promising strategy for patients with locally advanced pancreatic cancer. The influence of CPEC on therapeutic ratio of dFdC combined with radiation in pancreatic xenograft models is warranted for future clinical application.
